# The Medial Antebrachial Cutaneous Nerve Is a Low-Morbidity Alternative to the Standard Sural Nerve Autograft

**DOI:** 10.1177/15589447231218459

**Published:** 2024-01-05

**Authors:** Stahs Pripotnev, Sai L. Pinni, Suzanne Zhou, Gary Skolnick, Susan E. Mackinnon

**Affiliations:** 1 Western University, London, Ontario, Canada; 2 Washington University School of Medicine, St. Louis, MO, USA

**Keywords:** nerve graft, sural, MABC, autograft, nerve injury, donor site

## Abstract

**Background:**

Nerve interposition grafting is an important technique in nerve reconstructive surgery that is used when a primary repair is not feasible without significant tension. This study sought to evaluate the long-term morbidity of the medial antebrachial cutaneous (MABC) nerve as an alternative donor nerve in comparison with sural nerve harvest.

**Methods:**

A single surgeon and institution retrospective chart review was performed to identify all patients who underwent nerve autografting using the sural and MABC as donor nerves between January 1, 2000 and December 31, 2019. Surveys assessed overall patient satisfaction with surgery, as well as donor and recipient site morbidity, satisfaction, pain, numbness, and cold sensitivity.

**Results:**

Of the 73 patients contacted, 54 agreed to participate, and 43 of 73 (58.9%) ultimately completed the survey: 28 MABC (65.1%) and 15 sural (34.9%). There were no significant differences between the sural and MABC groups in overall satisfaction with surgery, donor and recipient site satisfaction, pain, cold sensitivity, and effect on quality of life. Even though 66.7% of sural donor sites and 75% of MABC donor sites had residual numbness, the effect this had on quality of life was very low (2 and 3, respectively).

**Conclusion:**

The MABC is a safe alternative to the traditional sural nerve autograft. A small subset of patients undergoing nerve autograft harvest will experience long-term morbidity in the form of pain. Conversely, the more common presence of numbness is not reported as bothersome.

## Introduction

Interposition grafting of upper-extremity nerve injuries is often required to perform a tension-free coaptation.^1-6^ Classically, the sural nerve has been the predominant source of nerve autograft donors.^7-11^ However, alternative options include the medial antebrachial cutaneous (MABC) nerve,^12,13^ lateral antebrachial cutaneous (LABC) nerve,^14,15^ posterior interosseous nerve (PIN),^16^ dorsal cutaneous branch of the ulnar (DCU),^17-19^ third web space of the median nerve (TWM),^20^ among others.^21^ Advantages of these alternative donors include confining donor morbidity to the affected limb, limiting additional incisions, simplicity of harvest, and the ability to repair the distal end of the cut nerve to the side of a normal nearby sensory nerve to minimize the pain and sensory loss of the harvested nerve.^21-23^ A major advantage of the sural nerve is the longest harvestable length at 30 to 50 cm compared with the MABC nerve (28 cm), LABC nerve (5-8 cm), PIN (2.5 cm), DCU (26 cm), and TWM (24.5 cm).^21^ The algorithmic approach to selecting a donor site has been previously described.^21^ This study sought to evaluate the long-term morbidity of these alternative donor nerves in comparison with sural nerve harvest.

## Methods

### Study Design

This study was approved by the Institutional Review Board of Washington University School of Medicine. A retrospective chart review was performed of a single surgeon’s practice at a single institution to identify all patients who underwent nerve autografting using the sural and MABC as donor nerves between January 1, 2000 and December 31, 2019. The study team contacted these patients in 2021 to 2022 to obtain long-term outcome data via surveys.

Chart review for patient characteristics at time of surgery included gender, date of birth, age, surgery date, smoking status, presence of diabetes, existing radiculopathy/plexopathy/other neuropathy, preoperative pain, date of surgery, recipient nerve, donor nerve, whether the surgery was a revision and/or required subsequent revision, and contact information.

### Survey Design

See supplemental content for full survey questionnaire (Supplemental Material S1). Survey data were collected using Qualtrics (Qualtrics, Provo, Utah). Patient demographics were obtained at time of survey, including age, sex, state of residence, hand dominance, and employment status.

Long-term postoperative outcomes included satisfaction with the overall reconstructive surgery and donor site. Surveys also asked about long-term donor and recipient site morbidity in terms of pain, numbness, and cold sensitivity. Pain presence, quality, intensity (visual analog scale [VAS] score), anatomical distribution, and effect on quality of life (QoL) were obtained. Patients who no longer reported pain also indicated how long it took for their pain to resolve. Similar outcomes were reported for numbness, including presence, anatomical distribution, quantified area (in^2^), qualitative area (comparison with golf ball, baseball, or football), effect on QoL, and time until resolution (if applicable). Questions about donor site morbidity were preceded by illustrations indicating donor site as a reminder and to prevent confusion between donor and recipient site.

Patients were contacted via phone call up to 3 times to obtain consent for participation in the study. Surveys were then distributed through the electronic medical record (EMR) and/or secure email links. Participants who did not have email addresses completed the survey over the phone. There was no compensation for participation.

### Statistical Analysis

Statistical analyses were conducted using Microsoft Excel 2022 (Version 16.58; Microsoft Corp., Redmond, Washington) for descriptive statistics and SPSS 2021 statistical software (Version 28.0.0.0; IBM Corp., Armonk, New York) for all other statistics. Statistical significance was defined as *P* < .05. Sub-analyses of all outcomes were also performed between the sural and MABC nerve autografts when they were used as a donor for a distant recipient to minimize overlap in donor and recipient site symptoms. Patients were included in the respective distant donor group if their recipient nerve was not located in the same extremity as their donor nerve harvest.

Shapiro-Wilk tests of postoperative outcomes showed multiple variables were non-normally distributed (<.001 ≤ *P* ≤ .585). Thus, for consistency, the distributions of all continuous variables are reported as median (interquartile range [IQR]), and Mann-Whitney *U* tests were used for between-group comparisons. Fisher exact tests were used to compare proportions between groups. Multivariate linear regressions were performed for 3 outcomes (pain intensity, effect of pain on QoL, and effect of numbness on QoL). Independent variables for all multivariate linear regressions were donor site, time since surgery, sex, and age at the time of surgery. In addition, multivariate logistic regressions with donor site and time since surgery as independent variables were performed to determine possible predictors of the presence of long-term pain, numbness, and cold sensitivity.

## Results

### Patient Demographics

Chart review identified 73 patients who were then contacted for study consent. Their procedures included 21 sural and 52 MABC. Eight patients refused the survey, and 11 were unreachable. Of the 54 patients who agreed to the survey, 43 (58.9%) completed the survey: 28 (65.1%) received MABC autografting and 15 (34.9%) received sural (Table 1). One patient who received donor nerve from both the MABC and TWM only completed a survey for MABC and was included in the analysis as an MABC patient. Recipient nerves included 5 accessory (11.6%), 1 facial (2.3%), 13 median (30.2%), 2 radial (4.7%), 14 ulnar (32.6%), 1 median/radial (2.3%), 1 median/radial/ulnar (2.3%), 1 plantar digital (2.3%), 3 peroneal (7.0%), and 2 sciatic (4.7%) (Table 2).

**Table 1.** Nerve Autograft Donor Site Survey Responses, Demographics, and Comorbidities.

Demographics	Sural nerve	MABC nerve	Total
Patients contacted, N	21	52	73
Declined participation, No. (%)	2 (9.5)	6 (11.5)	8 (11.0)
Unreachable, No. (%)	4 (19.0)	7 (13.5)	11 (15.1)
Agreed to participate, No. (%)	15 (71.4)	39 (75.0)	54 (74.0)
Survey responses, No. (%)	15 (71.4)	28 (53.8)	43 (58.9)
Age at surgery (average, range)	41 (27-51)	40 (22-54)	
Male, No. (%)	10 (66.7)	20 (71.4)	30 (69.8)
Female, No. (%)	5 (33.3)	8 (28.6)	13 (30.2)
Smoking, No. (%)	1 (6.7)	5 (17.9)	6 (14.0)
Diabetes, No. (%)	2 (13.3)	1 (3.6)	3 (7.0)
Neuropathy, No. (%)	0 (0.0)	0 (0.0)	0 (0.0)
Radiculopathy, No. (%)	0 (0.0)	1 (3.6)	1 (2.3)
Plexopathy, No. (%)	0 (0.0)	0 (0.0)	0 (0.0)

*Note.* MABC = medial antebrachial cutaneous.

**Table 2.** Recipient Nerves Grafted With Sural and Medial Antebrachial Cutaneous (MABC) Autograft Donor Nerves.

Recipient nerve, No. (%)	Donor nerve		
	Sural (n = 15)	MABC (n = 28)	Total (n = 43)
Accessory	0 (0.0)	5 (17.9)	5 (11.6)
Facial	1 (6.7)	0 (0.0)	1 (2.3)
Median	5 (33.3)	8 (28.6)	13 (30.2)
Radial	1 (6.7)	1 (3.6)	2 (4.7)
Ulnar	2 (13.3)	12 (42.9)	14 (32.6)
Median and radial	0 (0.0)	1 (3.6)	1 (2.3)
Median, radial, ulnar	1 (6.7)	0 (0.0)	1 (2.3)
Plantar digital	0 (0.0)	1 (3.6)	1 (2.3)
Peroneal	3 (20.0)	0 (0.0)	3 (7.0)
Sciatic	2 (13.3)	0 (0.0)	2 (4.7)

The ages of patients in the sural and MABC groups at the time of surgery were similar (sural: median [IQR] = 41.00 years [27.50-51.50] vs MABC: median = 40.0 years [22.50-54.25], *P* = .929). Most sural (n = 10, 66.7%) and MABC (n = 20, 71.4%) patients were men. There was 1 (6.7%) smoker at the time of surgery in the sural group and 5 (17.9%) in the MABC group. Two (13.3%) sural autograft patients had diabetes at the time of surgery, in comparison with 1 (3.6%) MABC patient. None of the sural patients had radiculopathy, plexopathy, or some other neuropathy, whereas 1 (3.6%) MABC patient had radiculopathy (Table 1). Patients treated with sural nerve autograft reconstruction were farther out from their surgery at the time of survey completion in comparison with the MABC group (13.15 years [10.69-15.04] vs 5.39 years [3.64-9.15], *P* = .003) (Table 3). Harvested graft length was not available.

**Table 3.** Comparison of Donor Site Satisfaction, Pain, Numbness, and Cold Sensitivity Between Sural and Medial Antebrachial Cutaneous (MABC) Autograft Donor Site Patients.

Donor site outcomes	Sural nerve	MABC nerve	*P* value
No. of patients, N	15	28	
Age at surgery (average, range)	41 (27-51)	40 (22-54)	.929
Years since surgery (average, range)	13.15 (10.69-15.04)	5.39 (3.64-9.15)	.003
Overall satisfaction (average, range)	8.00 (7.00-10.00)	8.00 (5.00-9.25)	.559
Donor site satisfaction (average, range)	8.00 (6.50-10.00)	7.00 (3.00-8.00)	.068
Donor site pain, No. (%)	2 (13.3)	12 (42.9)	.086
VAS score (average, range)	5.00 (4.50-5.50)	3.50 (3.00-5.00)	.352
Effect on QoL (average, range)	7.50 (6.75-8.25)	5.00 (2.75-7.00)	.270
Donor site numbness, No. (%)	10 (66.7)	21 (75.0)	.723
Numb area (in^2^) (average, range)	6.00 (5.00-15.38)	16.75 (12.00-30.00)	.144
Effect on QoL (average, range)	2.00 (0.00-4.00)	3.00 (2.00-5.00)	.233
Donor site cold sensitivity, No. (%)	2 (13.3)	11 (39.3)	.156

*Note.* MABC = medial antebrachial cutaneous; VAS = visual analog scale; QoL = quality of life.

### Preoperative Data

Due to the transition from paper charts to EMR during the chart review date range, preoperative pain scores were not available for most patients as this information was originally documented in paper form and was not adequately scanned into the EMR.

### Overall Satisfaction With Surgery

There were no significant differences between sural and MABC autografts for overall surgery satisfaction (sural: 8.00 [7.00-10.00] vs MABC: 8.00 [5.00-9.25], *P* = .559), which suggests that surgical outcome did not influence patient responses regarding their donor and recipient site symptoms (Table 3).

### Donor Site Satisfaction and Morbidity

There were no significant differences between sural and MABC autografts for donor site satisfaction (sural: 8.00 [6.50-10.00] vs MABC: 7.00 [3.00-8.00], *P* = .068). Sural and MABC groups did not have a significant difference in the proportions of patients who reported presence of pain (sural: 2 [13.3%] vs MABC: 12 [42.9%], *P* = .086), numbness (sural: 10 [66.7%] vs MABC: 21 [75.0%], *P* = .723), or cold sensitivity (sural: 2 [13.3%] vs MABC: 11 [39.3%], *P* = .156) (Table 3). Multivariate logistic regressions showed donor site and time since surgery were not significantly associated with long-term pain, numbness, or cold sensitivity.

Pain intensity also showed no significant difference between sural and MABC groups (sural: 5.00 [4.50-5.50] vs MABC: 3.50 [3.00-5.00], *P* = .352). There was no significant difference in effect of pain on QoL between sural and MABC groups (sural: 7.50 [6.75-8.25] vs MABC: 5.00 [2.75-7.00], *P* = .270). Effect of numbness on QoL showed no significant difference as well (sural: 2.00 [0.00-4.00] vs MABC: 3.00 [2.00-5.00], *P* = .233). Both groups were more likely to have numbness than pain (sural: 60.0% vs 13.3%, *P* = .011; MABC: 75.0% vs 42.9%, *P* = .017) (Table 3).

Multivariate linear regressions revealed donor site; age at the time of surgery, and sex were not significantly associated with pain intensity, effect of pain on QoL, or effect of numbness on QoL. Time since surgery was not significantly associated with pain intensity or effect of pain on QoL but was significantly associated with effect of numbness on QoL (*B* = −0.294, 95% confidence interval, −0.505 to −0.083, *P* = .008).

### Recipient Site Satisfaction and Morbidity

A follow-up round of surveys was sent approximately 1 year later to all patients who participated in the original survey to better delineate recipient site symptoms. Of the original 51 participants, 39 (76%) completed the follow-up questionnaire (18/28 [64%] MABC and 13/15 [87%] sural). The follow-up survey included the same questions as the original survey but instead focused on the recipient site rather than the donor site. There were no significant differences between sural and MABC autografts for patient age, recipient site satisfaction, recipient site pain, pain intensity, and pain effect on QoL (Table 4).

**Table 4.** Comparison of Recipient Site Satisfaction and Pain Between Sural and Medial Antebrachial Cutaneous (MABC) Autograft Donor Site Patients.

Recipient site outcomes	Sural nerve	MABC nerve	*P* value
No. of patients, N	13	18	
Age at surgery (average, range)	41 (33-53)	38.50 (20-53)	.674
Years since surgery (average, range)	13.13 (12.03-15.89)	5.17 (4.04-6.62)	<.001
Overall satisfaction (average, range)	8.00 (7.00-10.00)	8.50 (7.25-9.75)	.982
Recipient site satisfaction (average, range)	8.00 (5.00-10.00)	6.00 (2.25-8.00)	.115
Recipient site pain, No. (%)	5 (38.5)	12 (66.7)	.157
VAS score (average, range)	4.00 (2.00-7.00)	4.50 (3.50-6.00)	.915
Effect on QoL (average, range)	7.00 (2.00-10.00)	5.00 (4.00-8.00)	.790

*Note.* MABC = medial antebrachial cutaneous; VAS = visual analog scale; QoL = quality of life.

### Donor Site Satisfaction With Distant Recipient Sites

Ten patients in the sural group had distant recipient nerves, including 7 (70.0%) median, 2 (20.0%) ulnar, and 1 (10.0%) facial. The 6 MABC distant recipients included 5 (83.3%) accessory and 1 (12.5%) plantar digital nerve. There were no statistically significant differences between the sural and MABC group donor sites when they were used for distant recipients across all postoperative outcomes (Table 5).

**Table 5.** Comparison of Donor Site Satisfaction, Pain, Numbness, and Cold Sensitivity Between Sural and Medial Antebrachial Cutaneous (MABC) Autograft Donor Site Patients When Only Distant Recipient Sites Are Considered.

Donor site outcomes for distant recipients	Sural nerve	MABC nerve	*P* value
No. of patients, N	10	6	
Age at surgery (average, range)	49 (42-53)	35.50 (23-50)	.253
Years since surgery (average, range)	13.63 (10.75-17.64)	9.86 (6.66-16.23)	.329
Overall satisfaction (average, range)	8.00 (7.00-10.00)	9.00 (8.25-9.75)	.668
Donor site satisfaction (average, range)	8.00 (6.75-10.00)	8.00 (8.00-8.75)	.947
Donor site pain, No. (%)	1 (10.0)	3 (50.0)	.118
VAS score (average, range)	6.00 (6.00-6.00)	3.00 (2.50-3.00)	.157
Effect on QoL (average, range)	9.00 (9.00-9.00)	2.00 (1.50-4.50)	.180
Donor site numbness, No. (%)	7 (70.0)	5 (83.3)	1.000
Numb area (in^2^) (average, range)	6.75 (6.00-24.00)	9.25 (5.25-14.63)	.712
Effect on QoL (average, range)	1.00 (0.00-4.00)	2.00 (1.00-2.00)	.876
Donor site cold sensitivity, No. (%)	1 (10.0)	3 (50.0)	.235

*Note.* MABC = medial antebrachial cutaneous; VAS = visual analog scale; QoL = quality of life.

## Discussion

Nerve interposition grafting is an important technique in nerve reconstructive surgery that is used when a primary repair cannot be done without significant tension. It has been shown that as little as 15% strain in a tight nerve coaptation can cause a reduction in microvascular flow.^24^ Some common situations where grafting is required include patients with nerve injuries that present in a delayed fashion, patients with mechanisms that create a large zone of injury that must be excised, and also to bridge distances in nerve transfers.^1-6^

Although acellular nerve allografts (ANAs) have gained popularity in recent years, they are not economically available in many settings, have significant limitations, and in our experience are reserved for short sensory nerve reconstructions.^2,25-31^ Leckenby et al showed that in a series of 207 ANAs in 156 patients, meaningful motor recovery reduced from 67% with gap lengths of <3 cm to 38% with gap lengths between 3 and <5 cm and only 10% meaningful recovery with gap lengths >5 cm.^30,31^ Motor nerve deficits and sensory segments more than 4 cm are typically reconstructed with nerve autograft. In the literature, the sural nerve has historically been the most commonly selected donor nerve largely due to its ease of access, long length, and noncritical sensory donor deficit.^7-11^ Alternative nerve autograft donor sites such as the MABC, LABC, PIN, DCU, and TWM have advantages that include confining donor morbidity to the affected limb, limiting additional incisions, simplicity of harvest, and the ability to repair the distal end of the cut nerve to the side of a normal nearby sensory nerve to minimize the pain and sensory loss of the harvested nerve.^21^ In addition, with the increasing prevalence of obesity in the population, the positioning, dissection, and postoperative wound healing can be increasingly difficult at a sural nerve donor site. The purpose of this study was to compare the surgery satisfaction and donor site morbidity between these alternative donor sites and the classic sural nerve choice.

The long-term outcome questionnaire results showed that average overall surgery satisfaction was essentially identical between both groups which suggests that overall surgery outcome did not influence patient-reported symptoms and satisfaction with the donor site. Furthermore, the high overall satisfaction score supports the use of autografts when clinically indicated.

Although the MABC group had lower scores in donor site satisfaction, and higher prevalence of pain, numbness, and cold sensitivity in their donor site compared with the sural nerve group, these results were not statistically significant. A major confounding variable in the interpretation of these results is the proximity of the donor site and recipient site in the MABC group. Despite efforts to clearly delineate these 2 separate areas on the questionnaire, it is ultimately difficult for patients to differentiate their symptoms between donor and recipient when the reconstruction occurs on the same limb as the donor site. Thus, it is likely that patients reported lower satisfaction and worse symptoms due to overlap with recipient symptoms. Subgroup analysis using distant donor sites partially corrected for this confounder and the MABC started to trend toward improved satisfaction and lower symptoms, but this was also not statistically significant. In addition, despite the higher incidence of pain in the MABC group, the severity and effect on QoL was scored lower than the sural group which suggests that even if patients had difficulty differentiating between their donor and recipient site, the symptoms were less severe which supports the low morbidity of the MABC donor site in addition to the other advantages, including avoiding a second donor site.

Another potential confounding variable is the time since reconstruction. As the sural nerve patients were significantly further out from their surgical date, it is reasonable to assume that they would have had more time to recover and therefore have less bothersome symptoms. However, the multivariate linear regression showed no significant effect of time since surgery on the outcomes.

Although the purpose of the study was to compare alternative donor sites to the traditional sural nerve donor site, it is still important to note the overall incidence of these symptoms in either group at such a long-term follow-up. In the sural nerve group, at an average follow-up of 13.15 years, 13.3% of patients reported pain, 66.7% reported numbness, and 13.3% reported cold sensitivity. This is comparable and somewhat lower than the prevalence reported by Ducic et al who found that chronic pain occurred at a rate of 19.7%, sensory deficits at 92.9%, and sensory symptoms at 41.1% in a review article of 12 studies with shorter average follow-up periods.^10^ In addition, most other studies simply report the presence or absence of symptoms without quantifying the severity or impact of these symptoms. We found that although 13.3% of sural donor sites and 42.9% of MABC donor sites were reported as painful, the VAS pain score was rated as 5 and 3.5, respectively. The severity of the pain dropped even further to 3 for the MABC group when only distant donor sites were considered, and the confounding variable of the adjacent recipient site was eliminated. Similarly, even though 66.7% of sural donor sites and 75% of MABC donor sites had residual numbness, the effect this had on QoL was very low (2 and 3, respectively). This once again dropped even further to an effect on QoL of 1 (sural) and 2 (MABC) when only distant donors were considered. Our questionnaire found that pain more significantly affected QoL than numbness (7.50 [6.75-8.25] vs 2.00 [0.00-4.00]). This suggests that a small subset of patients undergoing nerve autograft harvest will experience long-term morbidity in the form of pain. Conversely, the more common presence of numbness is much less bothersome.

There are several limitations to this study. First, the study is retrospective and reviewed relatively small patient groups which may have made the results underpowered. This is even more prevalent in the distant recipient site subgroups. A prestudy power analysis was not completed. Second, the survey was done electronically with no follow-up clinical assessment. As a result, it is difficult to ensure patient understanding of the questions. Similarly, it was not possible to assess pain at rest compared with provocative maneuvers such as pressure over a neuroma. Third, certain surgical factors were not available such as the harvested graft length and the treatment of the proximal end of the harvested nerve. However, it is the principal investigator’s (SEM) practice to treat the proximal end by crushing the nerve away from the neurotomy site to set the location of regeneration more proximally, burning the neurotomy with cautery to cap it, and transposing the end into deeper tissue to place the potential neuroma further from the skin surface. More recently, another common practice for the principal investigator is to suture an allograft to the proximal end only to allow recovering axons to dwindle into the graft. Discussion regarding neuroma management is out of the scope of this article. Finally, although recipient site data were also collected, the variety in the location and type of reconstruction made it difficult to draw any specific conclusions from this component of the survey.

## Conclusion

Patients who underwent nerve grafting using the MABC had statistically similar outcomes and symptoms compared with those who received sural nerve autografts, including overall satisfaction with the surgery, donor site satisfaction, presence and severity of pain and numbness, effect on QoL, and cold sensitivity. Thus, the MABC is a safe alternative to the traditional sural nerve autograft and has multiple other advantages, such as confining the donor site to the same extremity, ease of harvest, and the ability to repair the distal end into the side of another nerve to minimize the sensory deficit. It should be considered as a source of nerve graft when the additional length of the sural nerve is not required.

## Supplemental Material

sj-pdf-1-han-10.1177_15589447231218459 – Supplemental material for The Medial Antebrachial Cutaneous Nerve Is a Low-Morbidity Alternative to the Standard Sural Nerve Autograft Supplemental material, sj-pdf-1-han-10.1177_15589447231218459 for The Medial Antebrachial Cutaneous Nerve Is a Low-Morbidity Alternative to the Standard Sural Nerve Autograft by Stahs Pripotnev, Sai L. Pinni, Suzanne Zhou, Gary Skolnick and Susan E. Mackinnon in HAND
