# Supplementary material for: The Medial Antebrachial Cutaneous Nerve Is a Low-Morbidity Alternative to the Standard Sural Nerve Autograft
Source: Hand (N Y). 2024 Jan 5;20(4):542–8. doi: 10.1177/15589447231218459 (PMC11571714; doi:10.1177/15589447231218459)
Supplement: sj-pdf-1-han-10.1177_15589447231218459 – Supplemental material for The Medial Antebrachial Cutaneous Nerve Is a Low-Morbidity Alternative to the Standard Sural Nerve Autograft [file sj-pdf-1-han-10.1177_15589447231218459.pdf]

Intro

You have been invited to participate in a research study because you had a nerve graft related surgery by Dr. [redacted]

A nerve graft involves taking a piece of nerve from one part of your body (the donor site) and using it at another part of your body (the recipient site) to reconstruct a missing function (strength or feeling), or decrease pain.

The purpose of this study is to understand the outcome at the area where the nerve graft was taken from (the donor site), and your overall satisfaction with this type of operation.

If you agree to participate, you will be asked to complete a short 10 minute electronic questionnaire. Although the questionnaire will initially collect your name, we will immediately de-identify the rest of your answers by assigning a study ID number instead of your name. You are free to not answer any questions that you prefer not to answer. You can choose to not participate by not answering the questionnaire or by notifying the study team that you changed your mind. If you partially or fully complete the questionnaire and then decide to withdraw your participation, you can contact the study team to delete your answers up until data analysis and publication.

The research results will not be returned to you after your participation.

There are no known risks from being in this study, and you will not benefit personally. However, other patients may benefit in the future from what we learn as a result of this study.

We will keep the information you provide confidential by assigning a study ID number to your answers. The file that connects your name to your study ID will be encrypted and password protected. It will be kept until the end of the study and then destroyed. However, federal regulatory agencies and [redacted] University, including the [redacted] University Institutional Review Board (a committee that reviews and approves research studies) and the Human Research Protection Office may inspect and copy records pertaining to this research. If we write a report about this study, we will do so in such a way that you cannot be identified.

You will not have any costs for being in this research study.

You will not be paid for being in this research study.

Your participation in this study is voluntary and you may choose not to take part. If you decide to participate in the study you may stop participating at any time. If you decide not to take part in the study or if you stop participating at any time, you won't be penalized or lose any benefits for which you otherwise qualify.

We encourage you to ask questions. If you have any questions about the research study, please contact: [redacted] If you have questions, concerns, or complaints about your rights as a research participant, please contact the Human Research Protection Office at [redacted] or email [redacted]. General information about being a research participant can be found on the Human Research Protection Office web site, [redacted]. To offer input about your experiences as a research

participant or to speak to someone other than the research staff, call the Human Research Protection Office at [redacted].

Thank you very much for your consideration. Returning the completed questionnaire will indicate your willingness to participate in the study.

Your nerve graft was taken from the outside of your ankle/leg. When the questions refer to the **"donor" site** or where the nerve graft was taken from, we are asking about the area on the outside of your foot and the outside of the bottom of your leg. This includes both the scar and the numb area beyond it. **Please see the following picture that shows the donor site highlighted in yellow.**

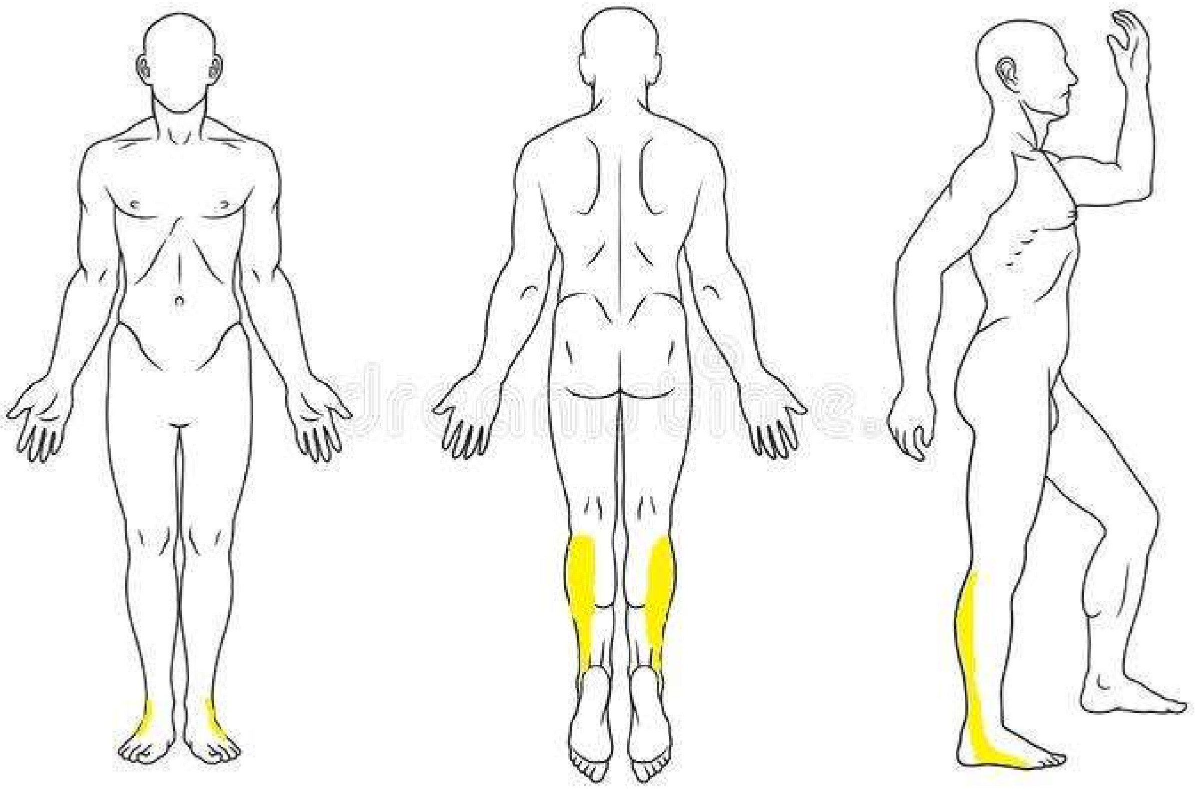

Demographics

What is your name?

What is your age?

What is your sex?

- Male
- Female
- Other
- Prefer not to say

What state do you live in?

What is your hand dominance?

- Right handed
- Left handed
- Ambidextrous (use both hands)

Do you remember having a nerve graft surgery?

- Yes
- No
- I'm not sure

Why did you have this surgery? **(select all that apply)**

- ☐ To decrease pain
- ☐ To increase sensation/feeling
- ☐ To increase movement/strength

Do you have a diagnosis of neuropathy (more than one area of numbness due to a medical condition)?

- ○ I'm not sure
- ○ No
- ○ Yes, I had this diagnosis before my nerve graft surgery
- ○ Yes, I got this diagnosis after my nerve graft surgery

Overall Satisfaction with Dr. [redacted] Surgery

How satisfied are you with the overall result of your nerve surgery from 0 (very dissatisfied) to 10 (very satisfied)?

Very dissatisfied							Very satisfied			
0	1	2	3	4	5	6	7	8	9	10

Reminder - Donor Site Refers to Issues in the Foot/Leg

The next sets of questions will ask you first about pain, and then numbness. They are all referring to the **donor site area which is on the side of your foot/leg**. This includes both the scar and the numb area beyond it. Please see the following picture as a reminder of the area in question.

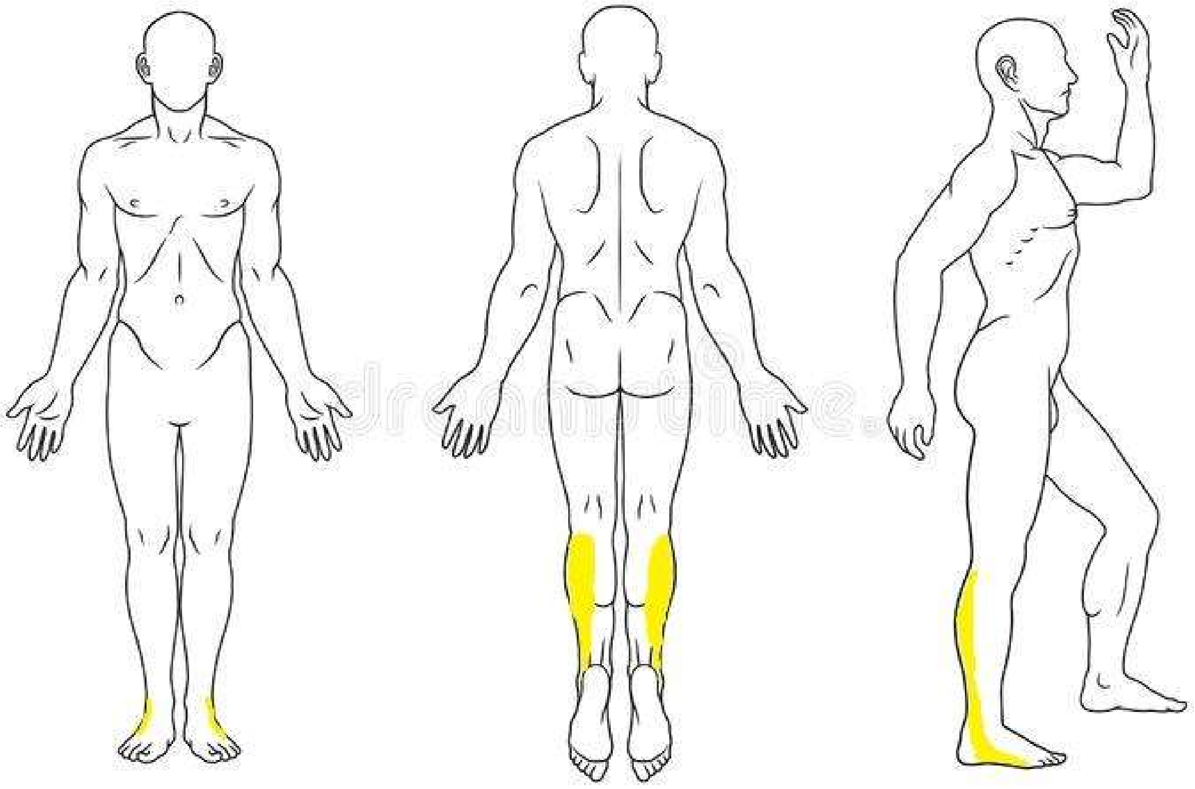

Donor Site Satisfaction

Overall, how much does your donor site **(side of your foot/leg)** bother you from 0 (not at all) to 10 (a large amount)?

		Not at all					A large amount			
0	1	2	3	4	5	6	7	8	9	10

Pain

Do you still have pain in your **donor site (side of your foot/leg)**?

- ○ No
- ○ Yes

Pain is difficult to describe. Click any words that describe your pain at your **donor site (side of your foot/leg)**.

- ☐ Burning
- ☐ Throbbing
- ☐ Aching
- ☐ Stabbing
- ☐ Tingling
- ☐ Twisting
- ☐ Squeezing
- ☐ Cramping
- ☐ Cutting
- ☐ Shooting
- ☐ Numbing
- ☐ Vague
- ☐ Stinging
- ☐ Indescribable
- ☐ Pulling
- ☐ Smarting
- ☐ Pressure
- ☐ Coldness
- ☐ Dull
- ☐ Itchiness
- ☐ Increased sensitivity
- ☐ None of these describe my pain

Slide bar to indicate your average level of pain at your **donor site (side of your foot/leg)** with zero being no pain and 10 being the worst pain you can imagine having.

No pain Worst pain

0 1 2 3 4 5 6 7 8 9 10

Where is your pain? Click on this diagram (up to 10 clicks possible).

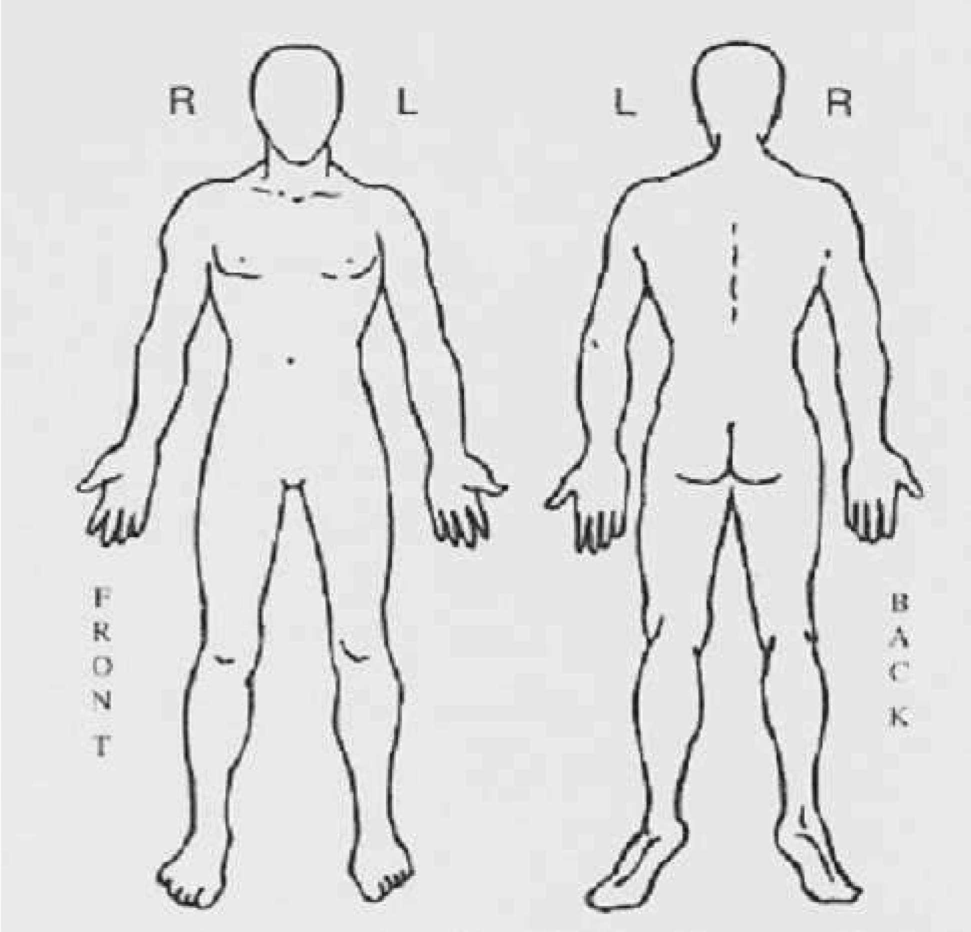

How much does the pain at your **donor site (side of foot/leg)** bother you from 0 (not at all) to 10 (a large amount)?

Not at all							A large amount			
0	1	2	3	4	5	6	7	8	9	10

If you no longer have pain at your **donor site (side of your foot/leg)**, how long did it take for the pain to go away?

- I don't remember
- Less than 1 month
- 1-2 months
- 3-5 months
- 6-11 months
- 1-2 years

- More than 2 years

Numbness

Do you still have numbness in your **donor site (side of your foot/leg)**?

- No
- Yes

Where is your numbness? Click on this diagram (up to 10 clicks possible).
Note: Pain will be marked on a separate diagram in a later question.

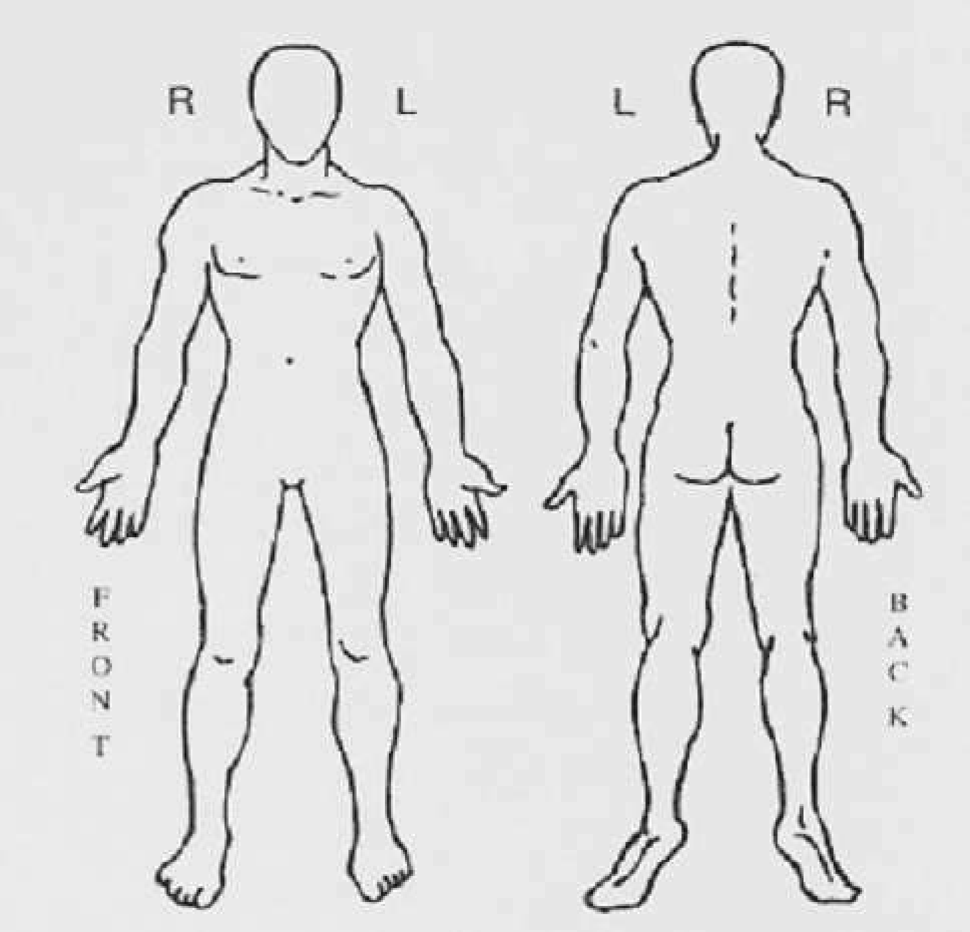

Please use a pen or marker to outline the area of numbness at your **donor site (side of your foot/leg)**. Use a ruler to measure this area in two perpendicular

dimensions. Please enter those measurements in the fields below. An example is provided below. (inches).

First dimension (inches)

Second dimension (inches)

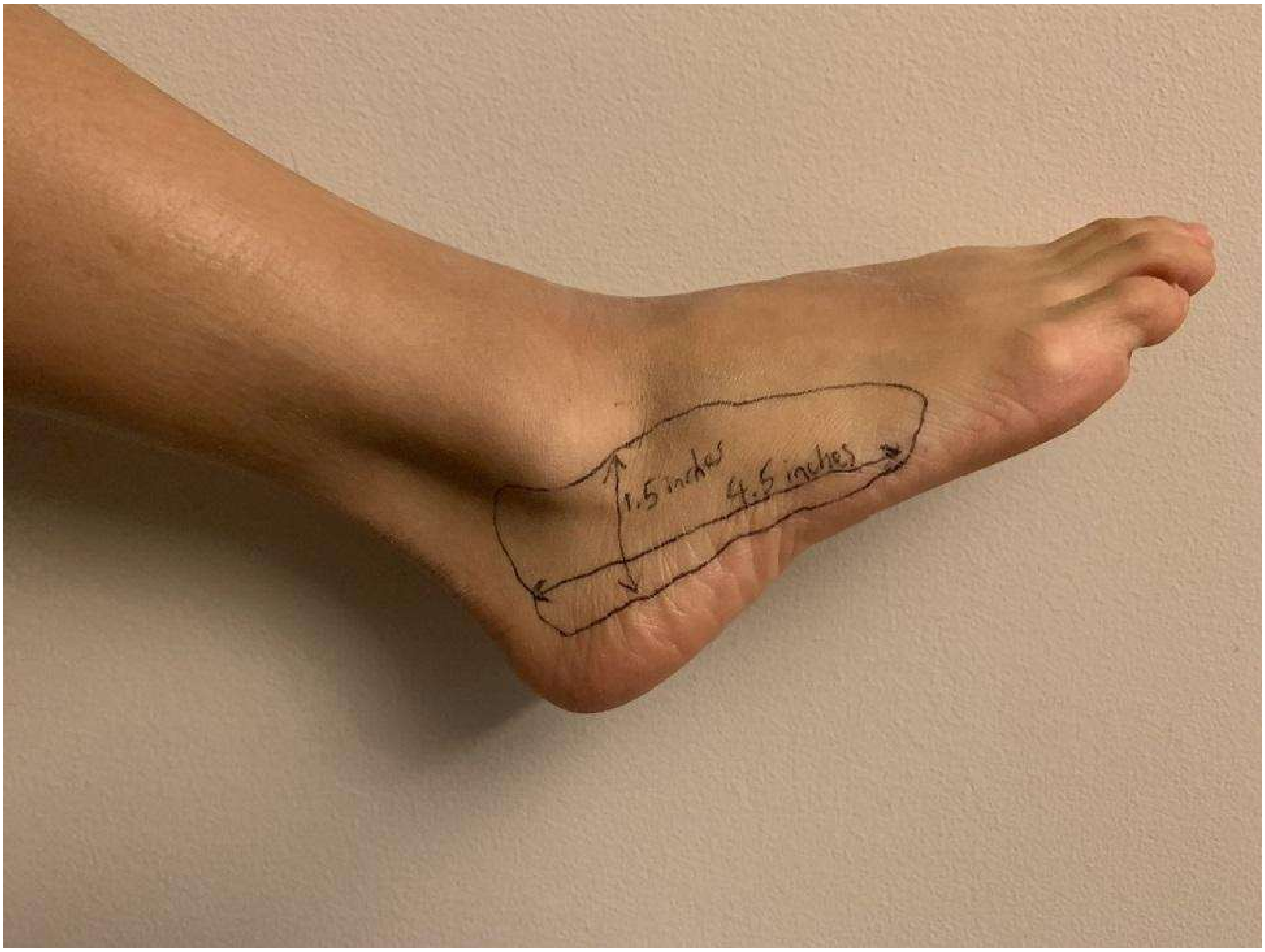

Which of the following items most closely estimates the size of the numb area at your **donor site (side of foot/leg)**?

- ◯ The size of a golf ball or smaller
- ◯ Larger than a golf ball up to the size of a baseball
- ◯ Larger than a baseball up to the size of a football
- ◯ Larger than a football

How much does the numbness at your **donor site (side of foot/leg)** bother you from 0 (not at all) to 10 (a large amount)?

Not at all						A large amount				
0	1	2	3	4	5	6	7	8	9	10

If you no longer have numbness at your **donor site (side of your leg/foot)**, how long did it take for the numbness to go away?

- I don't remember
- Less than 1 month
- 1-2 months
- 3-5 months
- 6-11 months
- 1-2 years
- More than 2 years

Cold Intolerance

Sometimes after an injury or surgery, the affected body part will experience cold sensitivity. Cold sensitivity is defined as increased pain, numbness, tingling, or weakness when the body part is exposed to cold temperatures. The following questions will ask you if you have increased symptoms when your **donor site (side of your foot/leg)** and hands are exposed to cold temperatures.

When your **donor site (side of your leg/foot)** is exposed to cold temperatures, do you have increased pain or other symptoms?

- I'm not sure

- No
- Yes

When your hand/arm is exposed to cold temperatures, do you have increased pain or other symptoms?

- I'm not sure
- No
- Yes, both hands/arms
- Yes, my right hand/arm
- Yes, my left hand/arm

Work

Are you currently? (select all that apply)

	No	Yes
Employed for wages	○	○
On medical leave	○	○
Homemaker	○	○
Self-employed	○	○
Student	○	○
Retired	○	○
Volunteer	○	○
None of the above	○	○

Are you able to do your household chores?

- Do the same level of household chores without discomfort
- Do the same level of household chores with discomfort

- ◯ Do a reduced amount of household chores
- ◯ Most household chores are now performed by others

Powered by Qualtrics
